# Laparoscopic-assisted small bowel resection for treatment of adult small bowel intussusception: a case report

**DOI:** 10.1186/1757-1626-1-432

**Published:** 2008-12-31

**Authors:** Donald Stewart, Michael Hughes, William W Hope

**Affiliations:** 1 South East Area Health Education Center, Department of Surgery, New Hanover Regional Medical Center, Wilmington, North Carolina, USA

## Abstract

**Background:**

Intussuception is a rare cause of intestinal obstruction in adults. Diagnosis is often difficult due to the variable and sometimes episodic nature of symptoms. Surgery is the recommended treatment option in adults if the diagnosis is proven.

**Case presentation:**

We present a case of a 33 year old Caucasian female admitted with a small bowel obstruction and no history of previous abdominal surgery. Patient did not improve with medical management consisting of bowel rest and nasogastric tube decompression. Surgery was consulted and patient was taken to the operating room for a laparoscopic-assisted small bowel resection for a small bowel intussusception caused by a submucosal fibroma.

**Conclusion:**

Our case highlights the feasibility and potential benefits of laparoscopy in assisting the diagnosis and treatment of small bowel obstructions.

## Background

Although fairly common in children, adult intussusception is relatively rare representing only 1% of patients with bowel obstructions [1,2]. Intussusception occurs when a proximal segment of bowel (intussusceptum) telescopes into the lumen of an adjacent distal segment (intussuscipiens) and can occur anywhere within the gastrointestinal tract. Unlike in the idiopathic nature of this process in children, most cases in adults have a demonstrable etiology which is found in 70% to 93% of cases [3-7] and is due to a malignant lesion in 52%[7].

Diagnosis of adult intussusception is difficult secondary to the variable symptoms that can be acute, intermittent, or chronic. Computed tomography has proven to be a valuable diagnostic tool, with an accuracy rate of 78% [7]. In adults, surgery is the recommended treatment secondary to the high rate of malignant lesions associated with this process. We report a case of a young female with a bowel obstruction secondary to a small bowel intussusception.

## Case report

**Figure 1:**
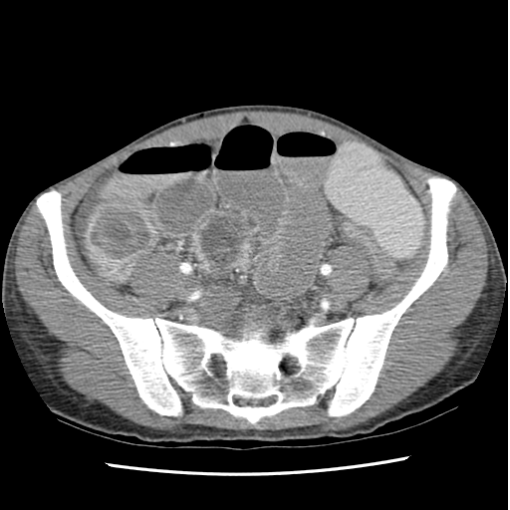
**Computed tomography scan showing dilated loops of small bowel consistent with a possible small bowel obstruction with possible target sign in right lower quadrant raising the possibility of intussusception**.

A 33 year old Caucasian female was admitted to the medicine service at our institution with an 18 hour history of abdominal pain, nausea, vomiting, and diarrhea. Patient had a strong family history of Crohn's disease including a sister that had onset of the disease at a similar age. Gastroenterology was consulted and a computed tomography scan was obtained showing edematous small bowel within the pelvis and a questionable internal hernia. Patient was treated medically for a small bowel obstruction including bowel rest, intravenous fluids, and nasogastric decompression without symptomatic relief. A repeat computed tomography scan was obtained four days after admission showing a possible transition point for her small bowel obstruction and mass like opacity possibly representing intussusception versus an internal hernia (Figure 1).

**Figure 2:**
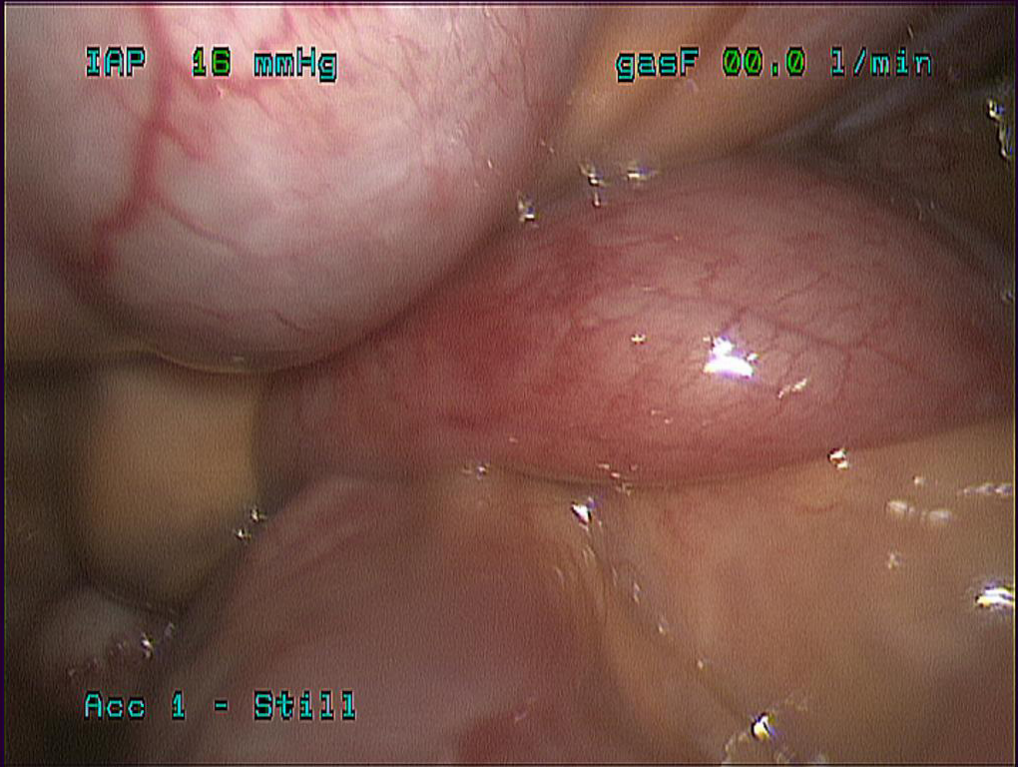
**Laparoscopic view of right lower quadrant just proximal to ileocecal valve**. Transition zone of small bowel obstruction is identified. Cause of obstruction is difficult to evaluate and we were unable to reduce laparoscopically.

**Figure 3:**
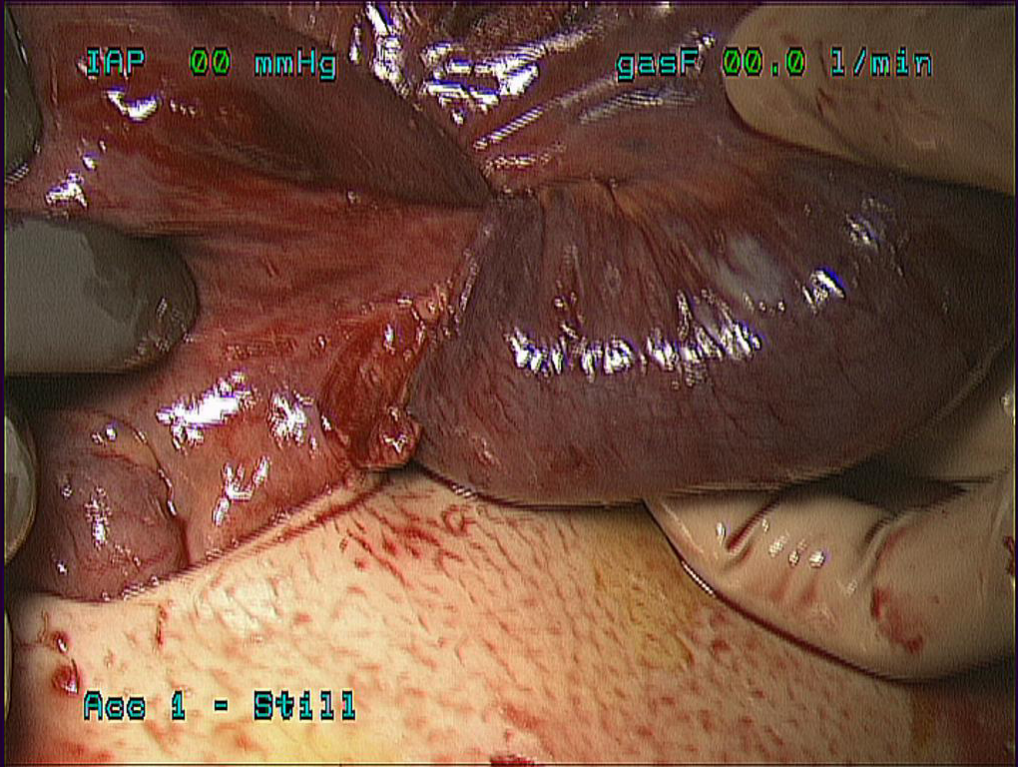
**Targeted infraumbilical incision has been made and small bowel and cecum brought out through the incision with clearly evident small bowel intussusception (ileo-ileal)**.

Surgery was consulted for evaluation and patient was counseled regarding risk, benefits, and alternatives of diagnostic laparoscopy, possible bowel resection, and laparotomy. The patient was taken to the operating room on hospital day five and underwent a diagnostic laparoscopy with specific attention to the right lower quadrant. Initial access was obtained via an open cutdown technique at the umbilicus. Three additional 5 mm ports were placed. Running of the small bowel was started at the cecum and progressed proximal from the ileocecal valve. Just proximal to the ileocecal valve an area of obstruction was identified, possible relating to an internal hernia (Figure 2). This area of obstruction was unable to be reduced laparoscopically so the cecum was mobilized laparoscopically. At this time a small 5 cm incision was made infra-umbilically and the cecum was elevated into the wound. A small bowel intussusception (ileal-ileal) was identified in the terminal ileum (Figure 3). The intussusceptum invaginated approximately 7 cm into the intussuscipiens and was partially reduced to allow a small bowel resection with an end to end stapled anastomosis approximately 6 cm from the ileocecal valve. Pathology revealed prolapsed small bowel consistent with intussusception with a well circumscribed firm tan-white submucosal nodule measuring 1.6 × 1.2 × 1.2 cm consistent with a submucosal fibroma.

**Figure 4:**
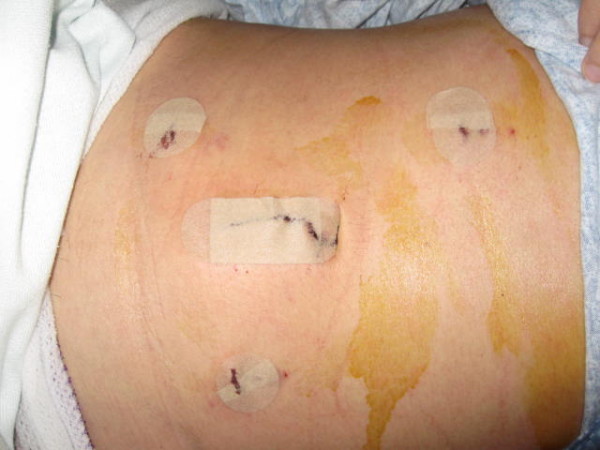
**Postoperative picture showing small infraumbilical targeted incision and 3 additional laparoscopic port sites**.

The patient tolerated the procedure well and was discharged home on postoperative day four with normal bowel function tolerating a regular diet. Patient was seen in follow-up doing well and without complaints (Figure 4.)

## Discussion

Surgery has been the mainstay of treatment for adult intussusception owing to the high association with malignancy as the underlying cause [7,8]. Abdominal exploration with resection of the involved section of bowel is usually recommended. With the advent of minimally invasive surgery and its expected benefits many general surgical procedures are now being performed or attempted laparoscopically.

Recently, minimally invasive techniques have been applied to the treatment of small bowel obstructions, specifically to the diagnosis and treatment of adult intussusception. Both laparoscopic and laparoscopic-assisted small bowel and colonic resections have been reported for both benign and malignant disease [9,10]. There are several attractive features of laparoscopy in the management of adult intussusception.

First, diagnostic laparoscopy may assist in the diagnosis of intussusception in cases such as ours where the diagnosis is suspected but not confirmed by preoperative workup. If the diagnosis is confirmed laparoscopically then appropriate surgical therapy and resection can be performed depending on the comfort level of the surgeon. Secondly, laparoscopy may aid in planning the incision if a laparoscopic-assisted or even laparotomy incision is required. In our case, we were unable to truly distinguish whether the obstruction in the right lower quadrant was due to intussusception or an internal hernia. However, the ability to mobilize the cecum and bring it up through a small periumbilical incision allowed for the appropriate diagnosis and ability to perform an extra-corporeal anastomosis. If laparoscopy had not been used in our case, then a much larger lower midline or even full laparotomy incision may have been needed to diagnosis and treat this distal small bowel intussusception.

## Conclusion

Our case highlights diagnostic laparoscopy and laparoscopic-assisted bowel resection as a potential and feasible tool in the treatment of small bowel intussusception. The ability to confirm diagnosis and plan targeted small incisions for treatment make laparoscopy a viable treatment option in patients suspected of having intussusception.

## Competing interests

The authors declare that they have no competing interests.

## Authors' contributions

DS and WH prepared the manuscript and reviewed the relevant literature. All authors were involved in clinical care and revising of the manuscript. All authors read and approved the final manuscript.

## Consent

Written informed consent was obtained from the patient for publication of this case report and accompanying images. A copy of the written consent is available for review by the Editor-in-Chief of this journal.
